# Educar para la salud en la Argentina de los años 1960: logros y balances del proceso de profesionalización de la educación sanitaria

**DOI:** 10.1590/S0104-59702025000100003

**Published:** 2025-02-14

**Authors:** Federico Rayez

**Affiliations:** i Becario doctoral, Consejo Nacional de Investigaciones Científicas y Técnicas; Universidad Nacional de Quilmes. Bernal – Provincia de Buenos Aires – Argentina. federicorayez@gmail.com

El libro *Educación sanitaria y desarrollismo, Argentina: 1960-1970*, de la investigadora argentina Carla Reyna, constituye un aporte relevante para comprender el proceso de profesionalización de la educación sanitaria en Argentina. La autora nos adentra en el mundo de las educadoras sanitarias argentinas en un momento crucial para esta ocupación: el pasaje a la profesionalización mediante la creación de una dependencia estatal a nivel nacional y cursos oficiales, así como también la creación de una revista especializada durante los años 1960.

La educación higiénica se ha desenvuelto como práctica y como campo de saber, con la finalidad de incidir y transformar, desde la instrucción y la propaganda, en los comportamientos sociales y culturales de la población. Como han demostrado varios trabajos (Ramacciotti, 2009; Gudiño Cejudo, 2012; Agostoni, 2015; Ferreira, 2023), la utilización de películas, carteles, folletos y otros materiales han sido importantes herramientas desplegadas para educar a las poblaciones de Argentina, México y Brasil en aras de que estas incorporaran valores, prácticas, saberes y precauciones que permitieran la prevención de enfermedades y un más alto nivel de salud.

El trabajo de la investigadora constituye un aporte fundamental en este sentido. En su primer capítulo, la autora nos presenta una de sus principales fuentes de archivo, la revista *Educador Sanitario*, cuyas recomendaciones analiza en los primeros tres capítulos. Esta revista fue producto de una renovación institucional, la creación de la Dirección de Educación Sanitaria y Social, en 1956, y tenía por finalidad promocionar la educación sanitaria como un campo de saber experto. Para esto, mediante un formato dinámico y moderno, difundía artículos sobre distintas problemáticas socio-sanitarias, con un fuerte acento en los problemas comportamentales, analizados desde la psicología, la psiquiatría y otros saberes expertos (Reyna, 2023). La meta era mostrar que las cuestiones sanitarias debían ser abordados con atención a las raíces culturales de esas “patologías”.

Los capítulos dos y tres están dedicados a un detallado análisis de temas y discursos de la revista. Según leemos en el segundo capítulo, la publicación sostuvo un fuerte interés en los tópicos relativos a la salud de niños, mujeres, jóvenes y familias de los estratos urbanos (Reyna, 2023). Le interesaba mostrar qué problemas tenían los y las jóvenes, cuáles eran sus conductas “patológicas” y cómo evitar que estos contrajeran enfermedades venéreas, toxicomanías o el alcoholismo. La clave estaba en trabajar los condicionamientos psíquicos, mejorar la comunicación entre padres e hijos y promover “un estilo de crianza moderno” centrado en “la contención emocional y la escucha atenta como recursos familiares indispensables para prevenir traumas infantiles” (p.37). Como vemos en el capítulo tres, las preocupaciones de la revista apuntaban al igual a la población marginal urbana, donde también veía un foco de intervención de la educación sanitaria.

El programa de la revista iba de la mano de las políticas de la Dirección de Educación Sanitaria y Social, dependencia en la que Reyna identifica un grupo de expertos muy dinámico compuesto por médicos, psiquiatras, educadoras sanitarias. Este es el tema del capítulo cuatro, en el que la autora analiza sus perfiles y cómo promovieron la formación de auxiliares en educación sanitaria. Este objetivo se habría empezado a realizar mediante la celebración de cursos en la Universidad Nacional del Litoral, en las Escuelas del Ministerio de Salud nacional y de la Provincia de Buenos Aires y en otros centros formativos desde principios de la década de 1960, siguiendo recomendaciones de la Organización Mundial de la Salud y otros organismos internacionales.

Un dato interesante aparece en el capítulo cinco del libro, en el que la autora explica los cambios producidos a partir del golpe de Estado de 1966. El gobierno cívico-militar, siguiendo una política de “subsidiariedad del Estado”, le otorgó una mayor importancia al Comité Argentino de Educación para la Salud de la Población. Esto implicaba darles mayor participación a asociaciones de la sociedad civil, en el entendimiento de que el Estado debía apartarse de aquellas tareas que podían ser desempeñadas por la comunidad, un tema ya tratado en trabajos de otros investigadores del período (Osuna, 2017).

El libro concluye con un balance en el que la autora indaga las disputas y conflictos profesionales que se fueron desarrollando en la década de 1960 y que concluyeron en un diagnóstico negativo. Como afirma Reyna, la educación sanitaria debió afrontar problemas y críticas provenientes de varios frentes. Desde la práctica de las educadoras, hubo críticas de otros profesionales de la salud sobre la idoneidad de la disciplina para lograr su cometido principal, el cambio de los comportamientos y representaciones culturales de la población. Desde la antropología y las ciencias sociales se afirmaba que la educación sanitaria carecía de un estatuto epistemológico y metodológico sólido. La Organización Mundial de la Salud advertía también que por el momento la especialidad era sólo un cúmulo desordenado de investigaciones y una serie de programas de compleja instrumentación.

Pese a esta nota negativa, los avances de la disciplina en el período fueron varios, como Reyna destaca a lo largo de la obra. La educación sanitaria cerró este período con un conjunto de temas apropiados y profundizados sistemáticamente en sus publicaciones expertas; logró poner en pie varias experiencias de formación de auxiliares y expertas; sentó las bases, finalmente, de una profesión duradera, que se expandió en las décadas siguientes.
